# Exploring Metro and Non-metro Differences in Satisfaction With Services and Community Participation Among Low-Income Personal Assistance Service Users

**DOI:** 10.3389/fresc.2022.876047

**Published:** 2022-06-02

**Authors:** Rayna Sage, Krys Standley, Genna M. Mashinchi

**Affiliations:** Rural Institute for Inclusive Communities, University of Montana, Missoula, MT, United States

**Keywords:** personal assistance services, personal care attendants, people with disabilities, rural, urban

## Abstract

Personal assistance services (PAS) are supports provided by workers to assist disabled people with their activities of daily living. Access to in-home PAS allows people who need assistance with these activities to live in their own homes and communities, rather than moving to congregate living facilities. Because metro and non-metro areas differ in many ways, we explored the following research questions: (1) Are there differences between non-metro and metro PAS users?, (2) What factors are associated with satisfaction with services?, and (3) What factors are associated with satisfaction with community participation?. We randomly surveyed PAS consumers in five states about their experiences with PAS. To answer the first question, we compared metro or non-metro consumers using independent samples *t*-tests. We found few statistically significant differences between metro and non-metro respondents. To answer the second and third research questions, we conducted linear regressions predicting our dependent variables. In terms of satisfaction with services, our model explained very little of the variance, other than finding that being partnered or married was significantly, positively related to satisfaction with services. In predicting satisfaction with community participation, the model explained about a quarter of the variance, with having fewer disabilities and higher health status predicting more satisfaction. This research indicates that there are few differences between metro and non-metro low-income PAS consumers and that more research is needed to understand what factors are related to satisfaction with services and community participation in this population.

## Introduction

For over ten million people with disabilities^1^, paid in-home support through Personal Assistance Services (PAS) (1) makes living and working in their community possible. These services provide critical assistance with activities such as bathing, dressing, toileting, housekeeping, and meal preparation (2). With adequate PAS, disabled people can remain in their homes and communities (3) and have the energy to comfortably and safely work, volunteer, socialize, and connect with others in their communities (3, 4).

Despite the importance of these services and the fact that work in PAS is one of the fastest growing occupations (5), little is known about how satisfied people are with these services, especially low-income people in non-metro areas. Furthermore, despite the importance of community participation in the well-being of people with disabilities (6), little research has explored how satisfied rural disabled people are with their community participation. This exploratory study considers differences between metro and non-metro PAS consumers and examines predictors of satisfaction with services and community participation. Here we present a brief overview of existing literature regarding satisfaction with services and community participation, followed by a summation of the need for more rural-specific research and related research questions.

### Satisfaction With PAS Services

There are a number of factors that have been explored in relation to satisfaction with PAS, but the majority have focused on issues around consumer choice and control in hiring, training, and maintaining their workers. Advocates during the Independent Living Movement in the 1970s and 1980s (7) pushed policymakers to find ways to move away from agency-controlled practices to consumer-directed models. Decades of research has established that consumer-directed programming is preferred over agency-based services (8, 9). Furthermore, some found high levels of satisfaction across different service delivery models, but elements of consumer choice and control (e.g., finding and hiring own aides, having the aide be a direct employee of the consumer, and more flexibility in who can be hired) were more related to satisfaction, regardless of the model (10). The most comprehensive research on the topic of self-directed models of care were related to the Money Follows the Person Demonstration Project (11), with research affirming that a move to more self-direction is associated with more satisfaction and less institutionalization. For example, across disability groups, moving out of institutions has been associated with more community participation and fewer barriers to community integration (12, 13). Despite challenges with community living, such as transportation barriers, Money Follows the Person beneficiaries have reported overall satisfaction with the program, including increased autonomy and overall well-being connected to living in their communities (14). Research on other models of care highlights how having more integrated services for consumers dually-enrolled in both Medicare and Medicaid led to higher levels of satisfaction with benefits and improved perceptions of quality of care (15). Finally, research into consumer characteristics such as race and gender of PAS users has found that Mexican Americans were more likely to have family caregivers than white consumers and that consumer race was not related to levels of satisfaction (16). Additionally, while satisfaction levels were similar across men and women, women were more likely to report problems with care.

### Community Participation

Having the ability to participate in community is a component of functioning related to health (17) that has become a standard for outcome measurement in rehabilitation (18) and can therefore be considered vital for the well-being of disabled people. Rural people face additional barriers in community participation related to transportation and limited services (19), which means using more time, energy, and resources than urban people to accomplish these activities. Adding to the complexity of the situation, rural people are generally older (20), more likely to be single and live alone (21), and have a higher rate of disability (21) than urban folks.

In short, compared to their urban counterparts, rural people with disabilities are doubly challenged in realizing their community participation goals because of higher levels of environmental barriers such as inaccessible infrastructure and a lack of public transportation (6). It is unknown how these various factors interact and potentially impact the community participation of disabled people who live in rural areas and rely on PAS. While little research exists regarding rural and urban differences in how PAS are delivered and used, there is reason to believe that rural PAS consumers may be less satisfied with their community participation experiences.

In this exploratory study, we used data collected in early 2020 (pre-pandemic) in a paper-and-pencil mail survey of PAS consumers. We examined differences in non-metro and metro PAS consumers and what factors are related to satisfaction with services and satisfaction with community participation in order to improve our understanding of this unique and understudied rural population. More specifically, we addressed three exploratory research questions:

1) Are there differences between non-metro and metro PAS users?
2) What factors are associated with satisfaction with services?
3) What factors are associated with satisfaction with community participation?

## Method

To answer these questions, we mailed a paper-and-pencil mail survey (copy of full survey available upon request) to 1,200 Consumer Direct Care Network PAS consumers in January of 2020. In addition to demographic questions and our variables of interest related to satisfaction with services and community participation, the survey also included questions on worker characteristics, health, electronic visit verification, and other topics relevant to PAS. We then conducted independent sample *t*-tests to explore metro and non-metro differences before constructing linear regression models to predict both satisfaction with services and community participation. To follow is a description of recruitment, measures, procedures, and analysis used.

### Recruitment

At the time of this study, our partnering organization (Consumer Direct Care Network) was serving mostly self-directed Medicaid-funded consumers in 17 states. Based on feedback from our Rural PAS Advisory Board (consisting of seven stakeholders including consumers, service providers, and organizational staff), we decided to target consumers in five geographically and programmatically diverse states: Arizona, Alaska, Montana, Texas, and Wisconsin. While the majority of consumers in these states were in self-directed programs that allowed for consumer-based worker recruitment, hiring, and management, some agency-based programming continues to exist. Thus, based on 5-year estimates from Consumer Direct Care Network administrative data, we sampled each state differently to maximize rural and agency-based representation (see Appendix A for more information).

### Procedures

After obtaining exempt status from the Institutional Review Board (IRB) at the University of Montana, we mailed pencil-and-paper surveys to 600 metro and 600 non-metro addresses across the five states. We used a Dillman multi-contact method (22), including a pre-notice letter, survey packet with $1 incentive, and follow-up postcard. Mailings were spaced approximately six days apart. Interested participants completed and returned the anonymous survey, which was expected to take 30 min. Research project staff were responsible for assembling the mailings and Consumer Direct Care Network staff applied mailing labels and mailed the materials to protect consumer confidentiality.

Of the 1,200 survey packets mailed out, 196 were returned because they were sent to undeliverable addresses, the person had died, or the person did not currently receive PAS. Surveys were returned by 190 participants, ten of which were omitted as they were completed by or for someone under the age of 18. This resulted in a response rate of 19%. Ninety percent of the respondents completed the entire survey. We received 96 non-metro and 85 metro responses.

### Measures

Relevant to this study, the survey included measures of basic demographics, disability type, general health status, satisfaction with services, satisfaction with community participation, metro/non-metro status, and service type (self-directed or agency). To follow is a description of each of the measures.

#### Demographics

In open-ended questions, participants were asked to indicate their gender and answers were categorized into women and men. Age was also asked using an open-ended question and dichotomized to be working age [18–65] and non-working age (66 and older). We measured race using a check-all-that-apply option of American Indian/Alaska Native, Asian, Black/African American, Native Hawaiian/Other Pacific Islander, White, and Other (with space for a write-in answer). We also asked participants to indicate if they identified as Hispanic or Latino. Answers were then collapsed into a single dichotomous variable of White, Non-Hispanic and Non-White or Hispanic. Partnered-status was measured by asking if participants were single/never married, single/divorced or separated, widowed, married, or living with a serious partner. Partnered-status was then dichotomized as married/partnered or single. To measure income, respondents were provided seven categories of income ranging from < $10,000 to $100,000 or more. For this analysis, these categories were collapsed into a dichotomous variable of $20,000 or less and $20,001 or more.

#### Disability Type

Disability type was measured using the six-item set of dichotomous questions which are also asked in the American Community Survey (23). Respondents were prompted to indicate if they: (1) are deaf or have serious difficulty hearing, (2) are blind or have serious difficulty seeing, (3) have serious difficulty concentrating or remembering, (4) have serious difficulty walking or climbing stairs, (5) have serious difficulty dressing or bathing, or (6) have serious difficulty doing errands alone. We constructed a count variable of disability by adding responses to these six questions together to indicate multiple disabilities.

#### General Health Status

General health status was measured using a single item from the Health Related Quality of Life Scale (24): “Would you say that in general your health is: poor, fair, good, very good, or excellent?”

#### Type of Care Provider

We asked respondents to answer the question: “If you have more than one paid caregiver, who provides the MOST assistance?”, to which they could select immediate family member, extended family member, friend or someone they knew before, someone they did not know, or fill in an “other” option. For this study, we dichotomized type of care provider as family (including family or extended family members) and non-family.

#### Satisfaction With Services

We used a 23-item modified version of the *Community Care for the Elderly (CCE) Client Satisfaction Survey* (25) converted to focus on PAS and added six related questions suggested by our Rural PAS Advisory Board members. The 23 items covered topics related to overall satisfaction, satisfaction with services, satisfaction with workers, and the impact of services on well-being, independent living, and community participation. The 23-item scale has a Cronbach's α of 0.92.

#### Satisfaction With Social and Community Participation

We used a four-item question set included in the PROMIS-29 scale (24) to indicate satisfaction with social and community participation. Respondents were asked to indicate on a five-point Likert scale (1 = never, 5 = always) how often they had trouble with: (1) doing all their regular leisure activities with others, (2) doing all the family activities they want to do, (3) doing all their regular work (including work at home), and (4) doing all the activities with friends that they want to do. Scores were reverse coded so that higher scores indicated more satisfaction. The 4-item scale has a Cronbach's α of 0.92.

#### Non-metro/Metro Status

Consumer Direct Care Network provided the research team with a list of de-identified zip codes of current self-directed and agency-based consumers. The zip codes were used to identify corresponding Rural-Urban Continuum Codes for each potential respondent. The research team created a cross-walk file based on guidelines provided by the Housing and Urban Development's Office of Policy and Development Research (26). This file was then used by Consumer Direct Care Network data analysts to assign non-metro and metro statuses. Non-metro status (Rural-Urban Continuum Codes = 4–9), based on the United States Office of Management and Budget's (OMB's) published standards that are applied to Census Bureau data, included any county with <50,000 people. Metro status was applied to Rural-Urban Continuum Codes of 1-3.

#### Program Type

As with non-metro/metro status, Consumer Direct Care Network data analysts matched consumer addresses with codes applied to the return envelopes to indicate self-direction or agency-based programming.

#### Statistical Analysis

We first used independent samples *t*-tests to compare non-metro and metro respondent characteristics and satisfaction with services and community participation to answer our first research question. We then tested two linear regression models to understand how geographic, demographic, program type, type of caregiver, multiple disabilities, and health status characteristics help explain differences in satisfaction with services and community participation to answer our second and third research questions.

## Results

### Non-metro and Metro Similarities and Differences

Table 1 includes means for each variable by non-metro and metro status and *p-*values resulting from independent samples *t*-tests indicate significant differences. All PAS users in this sample had very low-incomes, with two-thirds of metro and one in four non-metro respondents reporting household incomes of $20,000 or less. In general, the majority of respondents were unpartnered. The vast majority of respondents were utilizing self-directed services and non-metro respondents were significantly more likely to be self-directed than metro (96% vs. 82%, *p* < 0.01). Overall, the most common disability types were serious difficulties walking or climbing stairs, dressing or bathing, and running errands independently. Metro respondents were significantly more likely to report serious difficulty dressing and bathing compared to non-metro respondents (82% vs. 66%, *p* < 0.05), while non-metro respondents were significantly more likely to report serious difficulty running errands independently (93% vs. 50%, *p* < 0.05). Finally, metro respondents had significantly higher number of disabilities compared to non-metro (3.56 vs. 3.06, *p* < 0.05).

**Table 1:** Sample descriptive statistics by non-metro/metro status.

	**Non-Metro**	**Metro**	***p***
Women	66%	62%	0.603
Working age (18–65 years)	55%	62%	0.334
Married/partnered	10%	18%	0.167
White, non-hispanic	78%	66%	0.077
Household income $20,000 or less	76%	66%	0.166
Self-directed services	96%	82%	0.003
Paid family care provider	56%	54%	0.879
**Disability**			
Deaf	16%	22%	0.306
Blind/Low vision	19%	22%	0.614
Memory/Concentration	43%	57%	0.064
Walking/Climbing stairs	82%	80%	0.707
Dressing/Bathing	66%	82%	0.025
Running errands	93%	50%	0.028
Count of disabilities (mean, 0–6)	3.06	3.56	0.010
Health status (mean, 1–5)	2.48	2.32	0.311
Satisfaction with services (mean, 1–5)	3.96	3.85	0.259
Satisfaction with community participation (mean, 1–5)	2.65	2.37	0.263

### Linear Regression Results

Prior to conducting the linear regression analyses, we completed Pearson's correlations for all variables. There were no strong correlations to indicate the existence of interacting variables or multicollinearity. To follow is a brief summary of the linear regression results for both satisfaction with services and satisfaction with community participation.

#### Satisfaction With Services

Overall, the linear regression model (see Table 2) predicting satisfaction with services was not statistically significant. The only variable in the model that was significant in relation to satisfaction with services was being partnered, which was positively related (β = 0.586, SE = 0.233, *p* < 0.05).

**Table 2:** Linear regression predicting satisfaction with services.

	**β**	***SE***	***p*** **-value**	**VIF**
Non-metro	0.060	0.154	0.696	1.269
Women	0.231	0.156	0.143	1.200
Working age (18-65 years)	0.214	0.163	0.194	1.311
White, non-Hispanic	0.137	0.159	0.390	1.137
Household income < $20,000	0.080	0.189	0.672	1.648
Married/Partnered	0.586	0.233	0.014	1.531
Self-directed services	0.002	0.142	0.990	1.260
Paid family care provider	−0.028	0.063	0.662	1.068
Count of disabilities	0.269	0.234	0.254	1.349
Health status	0.002	0.142	0.990	1.545
Observations	88			
Adjusted *R*-Squared	0.06			
*F*-Value	1.54			

#### Satisfaction With Community Participation

The linear regression model (see Table 3) predicting satisfaction with community participation was statistically significant (*F* = 4.37, adjusted R-squared=0.26, *p* < 0.001), with the variables included in the model explaining 26% of the variance in satisfaction. In this model, the number of disabilities reported by the respondent was negatively related to satisfaction with services (β = −0.227, SE = 0.085, *p* < 0.01), while health status was positively related to the variable of interest (β = 0.371, SE = 0.118, *p* < 0.01).

**Table 3:** Linear regression predicting satisfaction with community participation.

	**β**	***SE***	***p*** **-value**	**VIF**
Non-metro	0.200	0.221	0.367	1.272
Women	0.075	0.222	0.736	1.205
Working age (18–65 years)	0.192	0.239	0.423	1.357
White, non-Hispanic	0.027	0.231	0.906	1.123
Household income < $20,000	0.132	0.272	0.628	1.736
Married/Partnered	−0.231	0.332	0.488	1.585
Self-directed services	−0.354	0.334	0.292	1.263
Paid family care provider	0.261	0.207	0.212	1.097
Count of disabilities	−0.227	0.085	0.009	1.271
Health status	0.371	0.118	0.002	1.506
Observations	96			
Adjusted R-Squared	0.26			
*F*-Value	4.37^***^			

*** *p < 0.001*.

## Discussion

In answer to our first research question, “Are there differences between non-metro and metro PAS users?”, we found that consumers of PAS in metro and non-metro areas were very similar. The exceptions were that non-metro consumers were more likely to be self-directed and have serious difficulties running errands independently compared to metro respondents, while metro respondents were more likely to have serious difficulties dressing and bathing and reported more disabilities on average than non-metro respondents. Higher rates of self-direction among non-metro consumers makes sense in that agencies, like many organizations and businesses, tend to operate out of and in urban centers.

These findings may point to barriers in the geographic or built environments of non-metro communities (e.g., lack of public transportation, ramps, or automatic doors in public and private buildings as a potential barrier to people with mobility limitations) (27). This seems particularly relevant in relation to the higher rates of serious difficulties running errands independently among non-metro respondents, perhaps highlighting how some disabilities are a product of the environment and not necessarily traits unique to non-metro individuals. The higher rates of serious difficulties dressing and bathing among metro respondents may be related to how some people with disabilities move from more rural places to more urban places for better access to services (28–30).

To answer our second research question, “What factors are associated with satisfaction with services?”, we found that our model was not effective in explaining variation in satisfaction. While we included variables indicated by previous research, only being partnered was significantly related to satisfaction with services. For the people in this study who are lower income and are receiving home-based services through Medicaid, rurality does not predict or relate to satisfaction with services, regardless of whether or not the care provider is a family member, or the type of program they are enrolled in.

To answer our third research question, “What factors are associated with satisfaction with community participation?”, we found that our model was effective in explaining some of the variance in satisfaction with community participation, but many demographic factors, including rurality, were not significantly related to this type of satisfaction. Instead, the number of disabilities experienced by respondents, as well as health status, seem to be driving the significance of this model. This appears to indicate that degree of functional impairment bears on people's satisfaction with community participation. Existing research highlights there are no differences in actual community participation between metro and non-metro older adults, but closer proximity to certain environmental features such as neighborhood resources and public transportation increased social participation across geography (27).

### Future Research Directions

Based on these findings, further research is warranted to better understand what factors are related to satisfaction with services and community participation. Although the 23-item measure of satisfaction with services had high reliability, a *post-hoc* factor analyses of the measure revealed that with more data, different aspects of satisfaction with services could provide a more nuanced understanding of PAS users' beliefs about the services they receive. Additional research is also needed to explore how PAS services might be organized or improved to overcome the unique environmental barriers in rural communities. Furthermore, additional research should seek to understand how to improve satisfaction with community participation for people with different disability types, especially disability related to mobility impairments and health status. Previous work suggests that pain, fatigue, and depression are negatively related to leaving the home (31) and thus, may also be important in satisfaction with community participation.

### Limitations

Strengths of this study were its use of several complementary measures of demographics, disability type, general health status, satisfaction with community participation, metro/non-metro status, and self-directed/agency-directed PAS. The study further benefitted from the investigators' efforts to evenly sample metro and non-metro PAS users. Because this study relied on self-report data, reporting bias was a potential limitation. Additional limitations included small sample size and missing data, both of which were connected to a low survey response rate. This response level suggests that PAS users are a challenging population to survey.

## Conclusion

The present study explored the differences between metro and non-metro PAS users and further examined factors that might contribute to satisfaction with services and satisfaction with community participation. Despite many differences between metro and non-metro locations and access to resources, our findings found very few differences between metro and non-metro PAS users. However, of note, the significant findings related to non-metro individuals having serious difficulty running errands and being more likely to have self-directed services are in line with past literature and underscore the difficulty non-metro users experience when attempting to access the resources they need, whether that be through a lack of services available or through a lack of accessible transportation. Additionally, based on our findings, accessibility and access might further play a role in community participation, as those with fewer disabilities and higher health status were more satisfied with community participation. Inasmuch, access to resources to help with health and disability status, which are disproportionately fewer in non-metro areas, might affect not only health status, but also the ability to connect with others in the community. Thus, bridging the gap in accessibility to resources such as transportation and services might not only facilitate the ability of non-metro individuals to meet their basic needs, such as running errands and having access to services, but also to engage in their communities.

## Data Availability Statement

The raw data supporting the conclusions of this article will be made available by the authors, without undue reservation.

## Ethics Statement

The studies involving human participants were reviewed and approved by University of Montana Institutional Review Board, University of Montana. Written informed consent for participation was not required for this study in accordance with the national legislation and the institutional requirements.

## Author Contributions

RS conceptualized and designed the study and analyzed the data and subsequently revised the draft and created the tables. KS wrote the initial draft of the manuscript. KS and RS drafted the appendix. GM provided critical review of the paper and suggested edits. RS, KS, and GM contributed to the review and final edits. All authors contributed to the article and approved the submitted version.

## Funding

This work was supported by the Research and Training Center on Disability in Rural Communities (RTC:Rural) under a grant from the National Institute on Disability, Independent Living, and Rehabilitation Research (NIDILRR; Grant Number 90RTCP0002-01-00), which is a Center within the Administration for Community Living (ACL), Department of Health and Human Services (HHS). The research does not necessarily represent the policy of NIDILRR, ACL, or HHS and one should not assume endorsement by the federalgovernment.

## Conflict of Interest

The authors declare that the research was conducted in the absence of any commercial or financial relationships that could be construed as a potential conflict of interest.

## Publisher's Note

All claims expressed in this article are solely those of the authors and do not necessarily represent those of their affiliated organizations, or those of the publisher, the editors and the reviewers. Any product that may be evaluated in this article, or claim that may be made by its manufacturer, is not guaranteed or endorsed by the publisher.

## Footnotes

^1^we use “people with disabilities” and “disabled people” interchangeability to reflect the preferences of current disability advocates.

## Supplementary Material

Supplementary Appendix A More in-depth details about the sampling strategy.

## Abbreviations

PAS: Personal assistance services
OMB: United States Office of Management and Budget.
