# Supplementary material for: Exploring Metro and Non-metro Differences in Satisfaction With Services and Community Participation Among Low-Income Personal Assistance Service Users
Source: Front Rehabil Sci. 2022 Jun 2;3:876047. doi: 10.3389/fresc.2022.876047 (PMC9397747; doi:10.3389/fresc.2022.876047)
Supplement: Supplementary Appendix A — More in-depth details about the sampling strategy. [file Data_Sheet_1.PDF]

Appendix A. Methods for over-sampling non-metro and agency-based consumers

We used stratified sampling in each state to capture programming and geography differences with 240 consumers being sampled from each state. We used five-year estimates of consumer characteristics based on program enrollment and zip code to stratify the samples. See Table 1 below for the estimates of the universe of consumers. Alaska only has self-directed programming. Non-metro and metro consumers were sampled at estimated rates estimated (46% and 54%, respectively). In Arizona, approximately 98% of the agency-based consumers are metro (n=607) and 2% are non-metro (n=12). For self-directed, it was estimated that 95% were metro (n=444) and 5% non-metro (n=23). We sampled the entire universe of non-metro in Arizona for both programs and proportionately sample the metro consumers based on the administrative data estimates of 71% agency-based and 29% self-directed (dividing the 85% left into 71% and 29%). Because there were more non-metro Montana consumers, we sampled the universe of agency-based consumers (estimates suggest non-metro=15, metro=86) and split the remaining sample between metro consumers based on estimates of 70% self-directed and 30% agency-based. For Texas (self-directed only), we will sample the universe of non-metro (estimated at 7%, n=144) and randomly recruit 96 metro consumers (approximately 5%). Finally, for Wisconsin (also self-directed only with a 20% non-metro estimate), we oversampled non-metro (60%, n=190) and sampled the metro consumers at 4% (n=50).

Table 1. Universe of consumers

Program type	AK	AZ	MT	TX	WI	Total
Self-directed	718	467	364	2062	1552	3739
Agency-based	0	619	102	0	0	721
Total	718	1086	466	2062	1552	5884

Table 2. Sampling estimates

	AK		AZ		MT		TX		WI		Total
	Self-directed	Agency-based	Self-directed	Agency-based	Self-directed	Agency-based	Self-directed	Agency-based	Self-directed	Agency-based	
Metro	110	0	60	145	97	42	96	0	50	0	600
Non-metro	130	0	23	12	86	15	144	0	190	0	600
Total	240	0	83	157	183	57	240	0	240	0	1200
